# Troubleshooting obstetric spinal anaesthesia at district hospital level

**DOI:** 10.4102/safp.v64i1.5529

**Published:** 2022-07-28

**Authors:** David G. Bishop, Simon P.D.P. le Roux

**Affiliations:** 1 Department of Anaesthesiology and Critical Care, Faculty of Health Sciences, School of Clinical Medicine, University of KwaZulu-Natal, Durban, South Africa; 2 Global Surgery Division, Department of Surgery, Faculty of Health Science, University of Cape Town, Cape Town, South Africa

**Keywords:** anaesthesia, resource-limited settings, emergency surgery, obstetric spinal anaesthesia, anaesthetic complications, caesarean section

## Abstract

Obstetric spinal anaesthesia is routinely used in South African district hospitals for caesarean sections, providing better maternal and neonatal outcomes than general anaesthesia in appropriate patients. However, practitioners providing anaesthesia in this context are usually generalists who practise anaesthesia infrequently and may be unfamiliar with dealing with complications of spinal anaesthesia or with conversion from spinal to general anaesthesia. This is compounded by challenges with infrastructure, shortages of equipment and sundries and a lack of context-sensitive guidelines and support from specialised anaesthetic services for district hospitals. This continuous professional development (CPD) article aims to provide guidance with respect to several key areas related to obstetric spinal anaesthesia, and to address common concerns and queries. We stress that good clinical practice is essential to avoid predictable, common complications, and hence a thorough preoperative preparation is essential. We further discuss clinical indications for preoperative blood testing, spinal needle choice, the use of isobaric bupivacaine, spinal hypotension, failed or partial spinal block and pain during the caesarean section. Where possible, relevant local and international guidelines are referenced for further reading and guidance, and a link to a presentation of this topic is provided.

## Introduction

A caesarean section (CS) remains the most performed operation globally; however, outcomes in low- and middle-income countries (LMICs) are significantly worse than in high-income countries. Whilst complication rates are two to three times higher, the mortality rate is 50 times higher in LMICs.^1^ Anaesthetic contribution to maternal mortality at CS accounts for one in seven maternal deaths,^2^ and in South Africa, over a third of anaesthetic deaths occur under spinal anaesthesia.^3^ In this continuous professional development (CPD) article, we aim to provide guidance on aspects of management of obstetric spinal anaesthesia, and in particular to address common concerns that arise at district-level hospitals. District hospitals, or Level 1 hospitals, provide generalist services from a range of clinical disciplines, and anaesthesia is commonly provided by generalist medical officers rather than dedicated anaesthetic practitioners.

## Good basic practice is key to avoiding complications

It should be self-evident that good preparation, good clinical practice and adherence to national guidelines will decrease the incidence and severity of anaesthetic complications. This is often difficult in LMICs, where one-third of hospitals do not have a reliable supply of electricity, three-quarters do not have pulse oximeters and a quarter do not have a reliable oxygen supply.^4^ Nevertheless, it is important that operating theatres are appropriately prepared, and attempts are made to meet a basic standard of care, including standard monitoring such as non-invasive blood pressure, oxygen saturation and electrocardiogram (ECG) monitoring. The capacity to administer general anaesthesia is an essential component of this care,^5^ and a functional anaesthetic machine is required as a minimum standard. This should include a reserve oxygen cylinder, functional suctioning apparatus and bag valve mask as a back-up.

Avoidance of drug errors is another key aspect of theatre practice. Tranexamic acid is currently recommended for use on diagnosis of post-partum haemorrhage.^6^ In parallel to its increased use, there has been an increase in inadvertent intrathecal administration of this agent.^7,8^ This is possibly linked to its similarity in appearance to bupivacaine and storage in close proximity to bupivacaine, either on the spinal trolley or on the anaesthetic drug trolley. This catastrophic drug error is often fatal, and we recommend that this drug be stored either in a locked drug cupboard or outside of theatre, in addition to meticulous drug-checking practices.

Safe obstetric spinal practice is outlined in the current Essential Steps in the Management of Obstetric Emergencies (ESMOE) guidelines, and this programme has been shown to improve knowledge and skills of trainees.^9^ This includes correct positioning with a right hip wedge in all patients, correct spinal dosing and appropriate fluid management.

## Are preoperative blood tests are required for spinal anaesthesia?

Laboratory tests are not routinely required prior to the administration of spinal anaesthesia, and these should only be performed where indicated by clinical conditions, such as preeclampsia or haemorrhage. Ward haemoglobin (Hb) should be carried out prior to surgery in all patients, and a full blood count (FBC) should be requested if the patient is anaemic (Hb < 10.0 g/dL). In an otherwise healthy parturient, HIV infection is not an indication for an FBC, as this does not confer increased risk of moderate thrombocytopaenia in the obstetric population.^10^

Whilst patients with severe preeclampsia should be referred to regional hospitals, many patients with mild preeclampsia are managed at district-level hospitals, and occasionally will progress to severe disease requiring urgent management at district hospitals. The National Guidelines for Hypertension in Pregnancy should be followed.^11^ All patients require an FBC in the preceding 6 h, and spinal anaesthesia may be administered with a platelet count > 75 000 and no other contraindications. If it is not possible to get a recent FBC, it is permissible to use results within 12 h. In the absence of blood count results and where there is urgency to carry out surgery, the choice of anaesthetic technique should be decided on an individual basis and ideally in consultation with the referral hospital’s anaesthetic department.

## Selection of needle type: Atraumatic versus conventional

**FIGURE 1:**
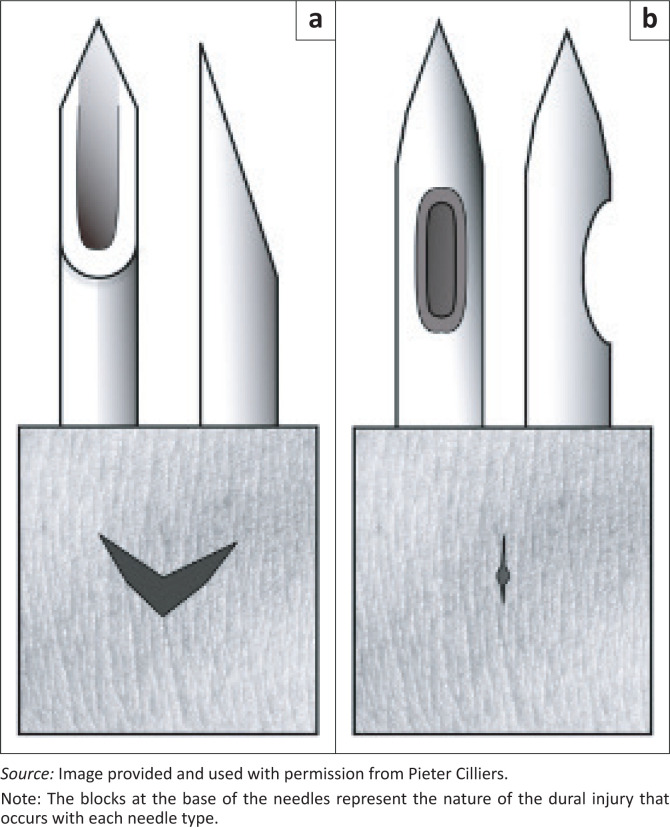
Conventional versus atraumatic needles. (a) Conventional; (b) Atraumatic.

Spinal needle type is important in order to reduce the incidence of postdural-puncture headache (PDPH). There is ample evidence that an atraumatic needle, such as the pencil-point needle, reduces the incidence of PDPH following obstetric spinal anaesthesia (risk ratio: 0.33, 95% confidence interval: 0.25–0.45),^12^ whilst the conventional ‘cutting’ needle (Quincke needle) has a high incidence of PDPH when compared with similar gauges.^13^ There is no difference in success rates between the two needles once practitioners become familiar with both techniques. It is possible that the Quincke needle is favoured for medical lumbar punctures in district hospitals and thus used when pencil-point needles are unavailable because of supply chain issues. However, even this practice should be examined, as the incidence of PDPH following lumbar puncture is 11% with conventional needles versus 4.2% with an atraumatic needle, without differences in success rates.^14^ The two needle types and the dural injury that occurs with each needle are illustrated in Figure 1. We recommend that it should be standard practice to primarily order atraumatic needles (25 gauge (G) or 26 G) for district hospitals, with cutting needles being reserved for rescue techniques or stock failures. The usual length of these needles is 90 mm; however, we recommend ordering a small quantity of extra-length needles (103 mm or 120 mm) for use in obese patients.

## Isobaric bupivacaine versus hyperbaric bupivacaine: Does it matter?

Current ESMOE recommendations support the use of 9 mg of 0.5% hyperbaric (‘heavy’) bupivacaine (1.8 mLs) in addition to 10 µg fentanyl (0.2 mLs) for routine spinal anaesthesia. This standard dose can be given regardless of the body mass index,^15^ but it may need to be reduced in patients of a very short stature (< 150 cm). However, drug shortages have become common, and district hospitals require a strategy when only isobaric (plain) bupivacaine is available. There is no evidence to support a difference between the two agents in terms of the height of block, the need for supplemental analgesia or conversion to general anaesthesia, although the quality of evidence is weak.^16^ The time to onset of isobaric bupivacaine might be slightly prolonged; however, either agent is acceptable and endorsed by the international consensus guideline.^17^ Effectively, there are two options available to clinicians:

- Use isobaric bupivacaine in the same dose as hyperbaric bupivacaine
- Mix hyperbaric bupivacaine by combining isobaric bupivacaine with dextrose (see Box 1 for an example, although several methods exist) and provide a slightly higher volume to achieve the same dose in milligrams.

BOX 1 Making Hyperbaric Bupivacaine

1. Ensure sterile or aseptic conditions
2. Draw up 4 mL 0.5% plain bupivacaine in a 5 mL syringe
3. Add 0.5 mL 50% dextrose to this, giving 4.5 mL total volume

This will constitute 4.5 mLs of 0.44% bupivacaine with 5.55% dextrose.

To administer 9 mg bupivacaine, 2.05 mL is required (give 2.0 mL)

To administer 10 mg bupivacaine, 2.27 mL is required (give 2.2 mL)

## Obstetric spinal hypotension: A common, serious complication

**FIGURE 2:**
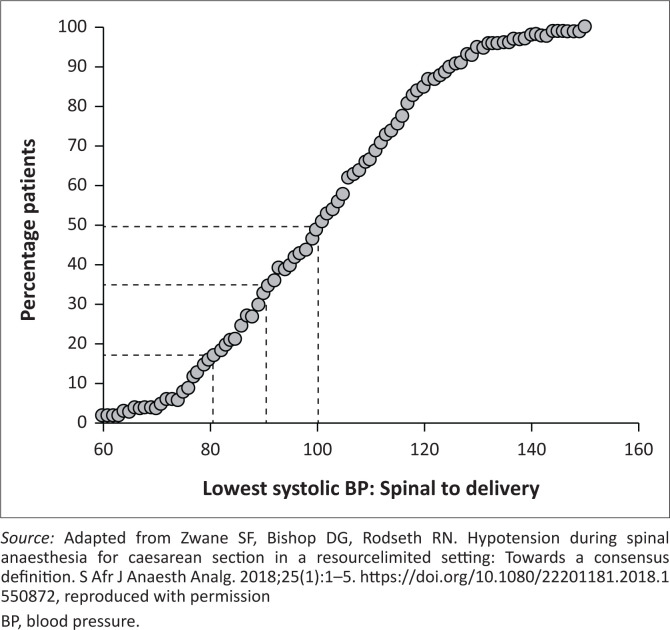
Incidence of obstetric spinal hypotension.

A recent international consensus guideline provides an excellent overview of this important topic, which specifically contains a section related to LMICs.^17^ Spinal hypotension is a common complication, and it should be anticipated and specifically prepared for in every patient. Half of the patients will experience a systolic blood pressure (SBP) below 100 mmHg, and almost one in five will experience an SBP below 80 mmHg (Figure 2).^18^

Spinal hypotension leads to poor placental perfusion and foetal compromise, maternal discomfort (including intraoperative nausea and vomiting) and if untreated may lead to maternal collapse. In South Africa, up to half of anaesthetic deaths have been ascribed to untreated spinal hypotension (NCCEMD National Anaesthetic Co-ordinator 2015, personal communication).^19^ The key to management is early and aggressive treatment, aiming at SBP > 90% of baseline blood pressure.^17^ This baseline blood pressure should be taken in the operation theatre immediately prior to spinal insertion. Treatment should include fluid therapy (especially if hypovolaemia is possible) and the use of either phenylephrine 50 µg – 100 µg (if heart rate > 60 beats per min [bpm]) or ephedrine 5 mg – 10 mg (if heart rate < 60 bpm) as boluses and repeated as necessary. Prophylactic phenylephrine infusions should also be considered and can be tailored according to the specific clinical context.^17,20^ If sufficient anaesthetic expertise is available, a titrated phenylephrine infusion, commencing at 50 µg/min and adjusting after 2 min is advised.^17^ If less anaesthetic expertise is available, a fixed-rate low-dose infusion (25 µg/min) is effective, simple to run and has been validated in a South African context.^21^ A simpler alternative is to add 500 µg phenylephrine to the first litre of Modified Ringer’s Lactate and run this over 10 min – 20 min once the spinal anaesthesia has been administered (which equals 25 µg/min – 50 µg/min).^22^ These recipes are outlined in Table 1. Additional bolus vasopressor therapy should also be administered if there is maternal nausea, an increased maternal heart rate (> 20% baseline) or a change in maternal level of consciousness. A low threshold for treatment is unlikely to cause harm and should be the default action when there is uncertainty. It is also important to realise that whilst phenylephrine is the vasopressor of choice for spinal hypotension (which is largely because of sympathectomy causing vasodilation), hypotension from other causes such as bleeding should be treated with fluids and either ephedrine or adrenaline as required.

**TABLE 1:** Suggested vasopressor management of spinal hypotension.

Anaesthetic provider	Phenylephrine infusion (50 µg/mL)
Experienced anaesthetist, no task-sharing	Initiate infusion at 50 µg/min, titrate after 2 min^17^ Aim for range 25 µg/min – 50 µg/min (30 mL/h – 60 mL/h)
Intermediate-level anaesthetist, no task-sharing	Fixed-rate low dose at 25 µg/min (30 mL/h)^21^ Additional boluses as required
Beginner anaesthetist or task-sharing	Put 500 µg in the first litre and run over 10 min – 20 min^22^ Additional vasopressor boluses as required

*Source:* Adapted from Kinsella SM, Carvalho B, Dyer RA, et al. International consensus statement on the management of hypotension with vasopressors during caesarean section under spinal anaesthesia. Anaesthesia. 2018;73(1):71–92. https://doi.org/10.1111/anae.14080

Whilst spinal hypotension is common, true high spinal anaesthesia is a rare, but potentially devastating complication. Local guidelines regarding the diagnosis and subsequent management have been developed, which should be specifically read by anaesthetic practitioners in South Africa.^23^ Warning signs include hypotension and bradycardia, weakness (especially of the hand grip), difficulty breathing and difficulty speaking. It is vital that early, aggressive treatment occurs, consisting of fluids and either ephedrine or adrenaline. Ventilatory support with mask ventilation and possibly intubation is important but follows vasopressor treatment. The recognition of patterns of hypotension should form part of training and are covered in guidelines referenced.^17,23^

## Failed or partial block at caesarean section

Block failure is a relatively common complication of neuraxial anaesthesia, although the exact incidence varies according to the definition and anaesthetic technique used. In the United Kingdom, a prospective audit suggested that 6% of patients do not have a pain-free operation after spinal anaesthesia, even though the dosing used was higher than in South Africa.^24^ Block failure approaches 10% in LMICs, with 22.8% requiring conversion to general anaesthesia and 23.1% requiring a repeat spinal anaesthetic in one study.^25^ Few guidelines exist related to a failed or partial block; however, the South African ESMOE guidelines state the following:

- If no effects after 20 min, repeat spinal injection
- If partial block, decide on:
  ■ Conversion to GA
  ■ Local anaesthesia with ketamine supplementation as required
  ■ Delay surgery for spinal later.
- If repeat injection has no effect, convert to general anaesthesia.

It is vital that there is a thorough assessment of the spinal block when following these guidelines. If there is any effect of the spinal anaesthetic, this must be considered a partial block and repeat injection is not advised, due to the risk of high spinal anaesthesia. These patients should either be postponed for 6 h or managed with a general anaesthetic if postponement is not possible.

## Pain during caesarean section

Pain during CS under neuraxial anaesthesia is now the leading cause of successful litigation in the United Kingdom.^26^ A South African study showed that more than a third of doctors surveyed were uncomfortable converting a spinal anaesthetic to general anaesthesia, despite being required to have this skill. Furthermore, only half of the anaesthetists routinely test the spinal block, and there was no agreement on how best to do this.^27^ Adequate testing of the block is a key component in avoiding intraoperative pain and will allow timeous repetition of the block or postponement of the case where possible. Testing the spinal block should include the following:

- Test using light touch, aiming for a level of T5. Check additionally with cold sensation if in doubt
- Test from blocked to unblocked, bilaterally, between the mid-axillary and mid-clavicular lines
- Test motor blockade using straight-leg raise, bilaterally.

Preoperative counselling should warn the patient that there will be some sensation of touch, but that pain should be reported. It is important to acknowledge the pain when it occurs and to stop surgery if possible. Consider the stage of the operation: significant pain prior to delivery of the baby is likely to require conversion to general anaesthesia. A stepwise management of pain should include consideration of the following options:

- Administer nitrous oxygen and oxygen (70:30 mix)
- Use fast-acting analgesic agents (e.g. fentanyl 25 µg – 50 µg intravenous injection [IVI])
- Ketamine boluses may be used in a titrated fashion (10 mg IVI)
- Consider local anaesthetic supplementation by the surgeon where appropriate
- Discuss conversion to general anaesthesia with the team and the patient.

Pain should not be treated with anxiolysis such as a benzodiazepine but with analgesic agents. Good communication is essential, and all patients who experience significant pain during the CS should receive postoperative counselling.

## Conclusion

Safe perioperative management of obstetric spinal anaesthesia is a core clinical skill for district hospital medical practitioners. It is important that existing guidelines are followed and that common complications are anticipated and planned for. A test for routine FBC should not be performed without a clinical indication. Atraumatic needles should be the default needle for spinal anaesthesia, and plain bupivacaine can be used in equivalent doses or mixed to make hyperbaric bupivacaine where there are drug shortages. Spinal hypotension should always be anticipated, and steps should be taken to manage this complication, with an emphasis on early vasopressor treatment. We also provide guidance on a failed or partial spinal block and the management of pain during the CS.

To access a presentation on this topic and other talks relevant to District Anaesthesia, use the following link: https://www.youtube.com/c/DistrictAnaesthesiaEducation
